# Editorial: Biomarkers in Genitourinary Cancers, Volume I

**DOI:** 10.3389/fonc.2022.965294

**Published:** 2022-07-08

**Authors:** Paula Alexandra Videira, Marco Borghesi, Eric A. Singer

**Affiliations:** ^1^ Research Unit on Applied Molecular Biosciences (UCIBIO) – Applied Molecular Biosciences Unit, Department of Life Sciences, NOVA School of Science and Technology, Universidade NOVA de Lisboa, Caparica, Portugal; ^2^ Associate Laboratory i4HB - Institute for Health and Bioeconomy, School of Science and Technology, NOVA University Lisbon, Caparica, Portugal; ^3^ Dipartimento di Scienze Chirurgiche e Diagnostiche Integrate, University of Genoa, Genoa, Italy; ^4^ Kidney Cancer Program, Rutgers Cancer Institute of New Jersey and Rutgers Robert Wood Johnson Medical School, New Brunswick, NJ, United States

**Keywords:** biomarkers, genitourinary cancer, bladder cacner, prostate cancer, renal cancer

Genitourinary cancers are known as significant causes of mortality worldwide. This heterogeneous group includes, among others, the most common cancer in men, prostate cancer, the most common form of kidney cancer, renal cell carcinoma (RCC), and the 10th most common cancer, bladder cancer. These entities present biological diversity with various histological subtypes and a poor prognosis when metastatic.

There has been considerable progress in treating patients with genitourinary cancers due to the improved understanding of their pathological mechanisms and the identification of meaningful biomarkers. The treatment progress has led to a fundamental paradigm shift in treatments. For example, our current understanding of the immunogenicity of these tumours has improved tremendously. Thanks to that, today, immunotherapy is a reliable strategy to improve the outcomes of patients with metastatic urothelial carcinoma, renal cell carcinoma, and prostate cancer. However, there is still a critical need to enrich our understanding of additional molecular mechanisms.

Along with the mechanisms, there is an urgent requirement to identify novel biomarkers to progress the diagnosis and prognosis of genitourinary cancers and their treatment. Biomarkers have become a significant focus of research, primarily on how they can help predict response to systemic therapy, identify treatment resistance, and avoid toxicities. Biomarkers that reveal the mutated tumour suppressor genes, the altered signalling pathways and the aberrantly expressed molecules help select potentially responsive patients to a given therapy. In this way, biomarkers improve outcomes and reduce costs related to ineffective treatments, and, most importantly, they significantly upsurge patients’ quality of life.

This Research Topic named Biomarkers in Genitourinary Cancers includes an interesting and up to date palette of publications from prominent research and clinical groups focused on identifying significant and emerging prognostic and predictive biomarkers. These biomarkers encompass non-coding RNA, serum proteins, gene expression, and glycans, among other entities identified in patients’ cohorts, samples and in the increasing number of public databases.

## Bladder Cancer

The review by Zhang et al. discussed biomarkers which predict bladder cancer lymphatic metastasis. The authors particularly emphasised the influence of non-coding RNA, its specific roles and prediction imaging models. In addition, they highlight non-coding RNA’s contribution to providing accurate diagnostic methods for future clinical applications⁠.

Wang et al. showed that among the non-coding RNA, the miR-20a-5p correlates with the recurrence of bladder cancer. Furthermore, the author reinforced that serum miR-17-92 cluster is overexpressed in bladder cancer. They also proposed a model composed of the three miR cluster members as a promising noninvasive biomarker for bladder cancer diagnosis⁠.

Li et al. constructed a prognostic signature to improve the prognosis prediction of advanced Bladder Urothelial Carcinoma based on ferroptosis-related genes. They used TCGA and another patient cohort, identified differentially expressed genes associated with overall survival, and generated a prognostic risk signature through LASSO regression analysis.

Xie et al. reported a novel model based on the ten inflammatory response-associated genes that can predict survival time for transitional bladder cancer. In addition, the authors provide clues for treatment strategies according to the drug sensitivity⁠.

Beyond genes, Carvalho et al. underscored the sialyl Tn as a cancer-associated glycan to detect urothelial bladder cancer cells in urinary samples that can serve as follow-up and long-term retrospective screening. In addition, the authors demonstrated that the microfluidic devices, which the authors called UriChip, can successfully be used to detect cancer cells in urine, paving the way for the development of a sialyl Tn -based medical devices.

## Prostate Cancer

Basourakos et al. highlight recent advances in using tissue-based genomic tests to select the best treatments for prostate cancer and the existing evidence supporting their clinical use⁠. Chiu et al. showed that the Prostate Health Index density (PHID), a diagnostic indicator calculated based on serum biomarkers and prostate volume is an efficient predictor of clinically significant prostate cancer (csPCa). Therefore, they suggested, the PHID risk table to be used in standard clinical practice to screen men at the highest risk of having csPCa.

Shi et al. explored The Cancer Genome Atlas (TCGA) and other public databases the Oncomine and found that in prostate cancer, lysophosphatidic acid receptor 1 (LPAR1) is positively correlated with chemokine/chemokine receptors, probably regulating the migration of immune cells. LPAR1 is a potential prognostic biomarker and plays an essential part in immune infiltrates in prostate cancer.

## Renal Cell Carcinoma

Kidney renal clear cell carcinoma (KIRC) and renal papillary cell carcinoma (KIRP) are the most common RCC types. Zhou et al. showed that the extracellular matrix protein collagen triple helix repeat containing 1 (CTHRC1) can predict tumour stage, metastasis and immune infiltration in KIRP and KIRC. This is due to the CTHRC1 role in modulating the tumour microenvironment and the authors also showed that its overexpression in KIRP and KIRC may be due to copy number variations (CNV) and DNA methylation⁠.

Li et al.’s performed a systematic review to investigate the prognostic value of aspartate transaminase (AST) to alanine transaminase (ALT) ratio, also known as De Ritis ratio. The authors concluded that De Ritis ratio significantly correlates with worse survival in patients with RCC. An elevated De Ritis ratio before treatments may serve as a prognostic biomarker in patients with RCC, although further studies are still necessary to validate this biomarker ⁠.

Cui et al. study suggested that Apolipoprotein C1 (APOC1) is a diagnostic and prognostic biomarker for clear cell Renal Cell Carcinoma (ccRCC). The author identified elevated APOC1 gene expression in databases and then, using tissue microarray, confirmed a significant correlation between APOC1, the tumour size and histological grade. Understanding the underneath tumorigenic mechanism may convert APOC1 into a new therapeutic target for the treatment of ccRCC.

On the other hand, Huang et al. analysed transcriptome data of ccRCC. They demonstrated that the ALDOB, EFHD1, and ESRRG genes are potential targets for medical therapy and could serve as diagnostic biomarkers for ccRCC⁠.

Zhu et al. investigated why ccRCC carrying wild-type Von Hippel–Lindau (VHL) tumour suppressor gene are more invasive and show higher morbidity. Applying applied bioinformatics approaches, these authors elected six survival-related differentially expressed RNAs upregulated in patients carrying wild type VHL, which helped to calculate risk scores predicting malignancy and prognosis⁠.

Studies conducted by Lv et al. highlighted the clinical significance of CD146 in ccRCC and provided novel insights into the immune function of CD146 in the tumour microenvironment ⁠. In another research article, the Lv et al. team showed that Fibrinogen-like Protein 1 (FGL1) facilitates the epithelial-to-mesenchymal transition (EMT) process and modulates the tumour microenvironment, which promotes ccRCC progression and metastasis. The authors suggest that targeting FGL1 can potentially improve the clinical outcomes of ccRCC patients⁠.

Zhang et al. put into evidence, in ccRCC, the role of pyroptosis, a programmed cell death with a highly inflammatory profile. The authors used gene expression data to show that pyroptosis regulators and pyroptosis index associated with the development and prognoses of ccRCC. Moreover, the authors validated the gene AIM2 the most significant immune-related pyroptosis regulator. AIM2 was therefore proposed as a predictor of the response to immunotherapy.

In non-metastatic RCC, ⁠Shang et al. demonstrated that neutrophil-associated NETosis and systemic lymphocyte perturbations occurred in patients with tumour thrombus and worse prognosis. This was replicated by a neutrophil-to-lymphocyte ratio ≥4 and considered an independent risk factor for patients ⁠. These two studies prove that assessing the cell death patterns may indicate the tumour status and guide therapeutic decisions. On the other hand, in metastatic RCC, Maruzzo et al. showed that thyroid hormones, when they reach a low fT3/fT4 ratio at baseline, are a decisive prognostic factor in patients under systemic treatment and independent of other biomarkers currently used in clinical practice⁠.

In two patients with metastatic ccRCC, and different sensitivity to axitinib–pembrolizumab combination Seront et al. found that in the case with decreased 68Ga metabolism it accompanies a decrease in size and number of lesions and, therefore a better response to treatment. The authors suggested that in ccRCC, 68Ga-Prostate-Specific Membrane Antigen (PSMA)-Positron Emitted Tomography (PET) predicts early response to systemic therapy. These studies confirm the relevance of PSMA as a predictive biomarker due to its significant expression in neovasculature⁠.

## Other Genitourinary Cancers ⁠

Nonaka et al. reported a rare case of Solitary fibrous tumours (SFT), which rapidly progressed to death after admission, which contrasted with SFT typical favourable prognosis. The authors screened for known mutation and gene expression. They reported the first evidence that mutations in the tumour suppressor gene TP53 mutations and downregulation of NAB2-STAT6 fusion gene expression associates with dedifferentiation of tumours and subsequent malignancy⁠.

In summary, this Research Topic summarised recent findings in the quest for reliable and meaningful biomarkers in genitourinary cancers. Such biomarkers are often identified based on large cohorts of patients and using computer-aided tools to guarantee new prognostic biomarkers, and promising therapeutic targets.

## Author Contributions

PV drafted the manuscript. ES revised the manuscripts. All authors made a substantial, direct, and indirect contribution to the work and approved it for publication.

## Funding

This work was also financed by national funds from FCT - Portugal, in the scope of the project UIDP/04378/2020 and UIDB/04378/2020 of the Research Unit on Applied Molecular Biosciences - UCIBIO and the project LA/P/0140/2020 of the Associate Laboratory Institute for Health and Bioeconomy - i4HB.

## Acknowledgments

We sincerely appreciate all submissions and contributing authors and reviewers for this Research Topic.

## Conflict of Interest

The authors declare the absence of any commercial or financial relationships that could be construed as a potential conflict of interest.

## Publisher’s Note

All claims expressed in this article are solely those of the authors and do not necessarily represent those of their affiliated organizations, or those of the publisher, the editors and the reviewers. Any product that may be evaluated in this article, or claim that may be made by its manufacturer, is not guaranteed or endorsed by the publisher.

